# Integrating Muscle Depletion with Barcelona Clinic Liver Cancer Staging to Predict Overall Survival in Hepatocellular Carcinoma

**DOI:** 10.3390/cancers17010024

**Published:** 2024-12-25

**Authors:** Tzu-Rong Peng, Chao-Chuan Wu, Jong-Kai Hsiao, Yi-Chun Chou, Yuan-Ling Liao, Yen-Chih Chen, Pei-Jung Shao, Ta-Wei Wu, Ching-Sheng Hsu

**Affiliations:** 1 Department of Pharmacy, Taipei Tzu Chi Hospital, Buddhist Tzu Chi Medical Foundation, New Taipei City 23142, Taiwan; tzu.rong@tzuchi.com.tw (T.-R.P.); tawei@tzuchi.com.tw (T.-W.W.); 2 Department of Surgery, Taipei Tzu Chi Hospital, Buddhist Tzu Chi Medical Foundation, New Taipei City 23142, Taiwan; ngchiukwan@yahoo.com.tw (C.-C.W.); oliverchen0308@gmail.com (Y.-C.C.); 3 School of Medicine, Tzu Chi University, Hualien 97004, Taiwan; swankyblade@yahoo.com.tw; 4 Department of Medical Imaging, Taipei Tzu Chi Hospital, Buddhist Tzu Chi Medical Foundation, New Taipei City 23142, Taiwan; jongkai@gmail.com; 5 Division of Gastroenterology, Department of Internal Medicine, Dalin Tzu Chi Hospital, Buddhist Tzu Chi Medical Foundation, Chia-Yi 62247, Taiwan; 6 Department of Chinese Medicine, Taipei Tzu Chi Hospital, Buddhist Tzu Chi Medical Foundation, New Taipei City 23142, Taiwan; ylliao.tcm@gmail.com; 7 Medical and Pharmaceutical Industry Technology and Development Center, New Taipei City 23142, Taiwan; pennyshao1992@gmail.com; 8 Division of Gastroenterology, Department of Internal Medicine, Taichung Tzu Chi Hospital, Buddhist Tzu Chi Medical Foundation, Taichung City 42743, Taiwan; 9 School of Post-Baccalaureate Chinese Medicine, Tzu Chi University, Hualien 97004, Taiwan

**Keywords:** hepatocellular carcinoma, sarcopenia, muscle depletion, noninvasive models, overall survival, prognosis

## Abstract

Background: Muscle depletion (MD) is a critical factor that influences clinical outcomes in patients with hepatocellular carcinoma (HCC). Although its role in cancer prognosis is recognized, its integration into widely used prognostic systems remains underexplored. This study aimed to evaluate the prognostic impact of MD on overall survival (OS) in HCC patients and to improve existing noninvasive prognostic models by incorporating MD-related metrics. Methods: A retrospective analysis was conducted on 1072 HCC patients treated at Taipei Tzu Chi Hospital between January 2006 and December 2016. All patients had follow-up data and computed tomography (CT) scans at vertebral level L3 for MD evaluation. Independent prognostic factors for OS were identified using Cox proportional hazards models, and the predictive performance of various prognostic models was assessed through the area under the receiver operating characteristic curve (AUROC). Results: The key independent predictors of OS in HCC patients included hepatitis B virus infection, hepatitis C virus infection, liver cirrhosis, tumor size, serum alpha-fetoprotein levels, and MD-related metrics (psoas muscle-to-spine ratio, psoas muscle-to-vertebral ratio, and myosteatosis). Among existing models, the Barcelona Clinic Liver Cancer (BCLC) stage, the Child–Turcotte–Pugh (CTP) class, and the albumin–bilirubin (ALBI) grade demonstrated robust predictive performance for OS. However, incorporating MD significantly improved the predictive accuracy of these models, with the MD–BCLC model showing the highest AUROC (0.804, 95% CI: 0.777–0.832, *p* < 0.001). Conclusions: MD is an independent and significant prognostic predictor for patients with HCC. Integrating MD metrics into established systems, particularly the BCLC staging system, markedly improves OS prediction, providing a more comprehensive tool for clinical decision-making in the management of HCC.

## Simple Summary

Muscle depletion (MD), a key predictor of survival in hepatocellular carcinoma (HCC), is often excluded from current prognostic tools. Analyzing data from 1072 HCC patients at Taipei Tzu Chi Hospital (2006–2016), this study identified MD-related factors, along with hepatitis and tumor size, as significant survival predictors. Incorporating MD into the Barcelona Clinic Liver Cancer (BCLC) staging system improved survival prediction accuracy, offering a more reliable tool for HCC management and better clinical decision-making.

## 1. Introduction

Hepatocellular carcinoma (HCC) is the leading cause of cancer-related mortality worldwide. It accounts for approximately 80% of primary liver cancer cases [1,2]. Patients with early-stage HCC and preserved liver function can achieve a 5-year survival rate of up to 70% after tumor resection [3]. However, the prognosis for most patients with HCC remains poor. In cases of unresectable HCC, the median survival time is less than one year [4]. These challenges highlight the urgent need to identify reliable prognostic factors and develop comprehensive risk stratification systems to improve the management and outcomes of HCC.

The survival of patients with HCC is influenced by a variety of factors, including age, tumor characteristics (such as size, number, vascular invasion, and extrahepatic spread), serum alpha-fetoprotein (AFP) levels [5], physical condition, and liver functional reserve. Liver reserve is commonly assessed using metrics such as the Child–Pugh class, bilirubin and albumin levels, clinically significant portal hypertension, and ascites [6]. To better evaluate patient risk, several prognostic staging systems have been developed based on these factors. Among these, the Barcelona Clinic Liver Cancer (BCLC) staging system is the most widely used worldwide [7,8], with extensive external validation across various clinical settings. However, the BCLC system relies on the Child–Pugh classification to assess liver reserve, which is limited by subjective criteria and suboptimal predictive power, thereby restricting its clinical utility. To address these limitations, newer noninvasive models and parameters for liver functional reserve assessment have been introduced. Notably, sarcopenia, the loss of skeletal muscle mass and strength, has emerged as an independent predictor of overall survival (OS) and progression-free survival in HCC patients, offering a promising avenue for enhancing prognostic accuracy.

Sarcopenia is a progressive, generalized skeletal muscle disorder characterized by the loss of muscle mass, strength, and function [9,10]. A specific manifestation of sarcopenia, muscle depletion, can be quantified using computed tomography (CT) and is associated with an elevated risk of complications such as hepatic encephalopathy and infection, prolonged hospitalization, increased healthcare costs, higher mortality rates, and poorer prognoses in patients undergoing HCC treatments [11,12]. Given these implications, we hypothesize that incorporating muscle depletion into existing survival prediction models could enhance their prognostic accuracy for patients with HCC.

In this study, we evaluated the prognostic significance of muscle depletion alongside 14 noninvasive liver reserve models for predicting overall survival (OS) in HCC patients. Additionally, we developed new survival prediction models by integrating muscle depletion into commonly used HCC prognostic systems. The performance of these enhanced models was then compared to that of traditional models to assess their predictive accuracy for OS in patients with HCC.

## 2. Methods

### 2.1. Ethics Statement

This study was conducted according to the principles of the Declaration of Helsinki and was approved by the Ethics Committee of Taipei Tzu Chi Hospital, Buddhist Tzu Chi Medical Foundation (12-X-098). The requirement for written informed consent was waived because this study was a retrospective analysis.

### 2.2. Study Design and Patient Selection

This study is a retrospective analysis of a prospective cohort of patients with hepatocellular carcinoma (HCC) who received various treatments. HCC was diagnosed through a combination of blood tests, AFP levels, chest radiography, abdominal ultrasound, pathology, dynamic contrast-enhanced CT, and MRI, as per international guidelines [6]. We enrolled patients aged ≥ 18 years with a new diagnosis of HCC, followed up at the gastroenterology clinics of Taipei Tzu Chi Hospital from January 2006 to December 2016. Only patients with available CT images at the L3 spine level for muscle depletion assessment were included. Exclusion criteria included patients lacking essential baseline data for the noninvasive liver reserve model evaluation.

### 2.3. Data Collection

We obtained patients’ baseline clinicodemographic data (i.e., recorded at the time of diagnosis), including age, sex, date of HCC diagnosis, tumor–node–metastasis classification, BCLC stage, Child–Turcotte–Pugh (CTP) score, liver cirrhosis status, hepatitis B virus (HBV) and hepatitis C virus (HCV) status, serum biochemical analysis results, and imaging data. We excluded patients without the adequate baseline data required for the assessment of noninvasive liver reserve models. Serum biochemical analysis results, liver function tests, AFP level, and abdominal ultrasound were performed every 3–6 months. Every 8–12 weeks, a tumor assessment was conducted using dynamic contrast-enhanced CT according to the modified Response Evaluation Criteria in Solid Tumors (mRECIST). The main outcome was OS, which was defined as the duration from the date of HCC diagnosis to the date of any cause of death, the date of loss to clinical follow-up, or the data cutoff time. The data of patients who were lost to follow-up were censored on the last date the patient was known to be alive, and patients who remained alive were censored at the time of data cutoff (30 October 2022).

### 2.4. Grading of Noninvasive Liver Functional Reserve Models

The 14 noninvasive liver functional reserve models were calculated using baseline clinical and biochemical data [4,13,14,15,16,17,18,19,20]. Receiver operating characteristic (ROC) analysis was used to determine optimal thresholds for MD diagnosis, focusing on metrics such as the psoas muscle-to-spine ratio, the psoas muscle-to-vertebra ratio, and the CT density of the psoas muscle area. Among these, the psoas muscle-to-spine ratio demonstrated superior performance according to the corrected Akaike information criterion (AICc). The area under the receiver operating characteristics curve (AUROC) indicated that the optimal cutoff to define muscle depletion was a psoas muscle-to-spine ratio of 4.42 (AUROC: 0.641, 95% CI: 0.607–0.675, sensitivity: 0.641, specificity: 0.566), a psoas muscle-to-vertebra ratio of 2.32 (AUROC: 0.602, 95% CI: 0.566–0.637, sensitivity: 0.602, specificity: 0.54), and a CT density of a Psoas muscle area of 37.2 (AUROC: 0.577, 95% CI: 0.541–0.613, sensitivity: 0.577, specificity: 0.532). Accordingly, muscle depletion was defined as a psoas muscle-to-spine ratio of <4.42. The liver functional reserve was graded from 1 (adequate liver function) to 3 (poor liver reserve; Table 1). Table 2 presents a new prognostic scoring system that incorporates muscle depletion with other models of noninvasive liver functional reserve.

### 2.5. Assessment of Muscle Depletion

In a previous prospective study of the psoas muscle-to-spine ratio predicting overall survival in patients with hepatocellular carcinoma, we performed abdominal CT scans using Lightspeed Pro 16 and Lightspeed VCT devices from GE Healthcare, Milwaukee, WI, USA in patients with HCC.

The Lightspeed Pro 16 protocol included the following: a tube voltage at 120 kV, a mA modulation technique (noise index of 11 with a mA range of 150–550), a gantry rotation time of 0.8 s, a pitch of 0.984, a collimation of 1.25 mm, a reconstruction slice thickness of 5 mm, a post-injection delay time of 70 s, and a window width/level of 350/45. Conversely, the Lightspeed VCT protocol consisted of the following: a tube voltage at 120 kV, a mA modulation technique (noise index of 11 with an mA range of 150–550), a gantry rotation time of 0.8 s, a pitch of 0.984, a collimation width of 1.25 mm, a reconstruction slice thickness of 5 mm, a post-injection delay times of 70 s and 35 s, and window widths/levels of 350/45 and 400/40. We captured non-contrast enhanced abdominal images of the patients at the L3 level. Two trained CT readers used Image J software (v1.54) to measure the total area of the right and left psoas, erector spinae, quadratus lumborum muscles, and the area of the lumbar vertebral body, with the complete transverse process, the spinous process, and the largest area of the vertebral foramen in the same CT plane. Muscle depletion was assessed by calculating the total psoas muscle area (the total area of the psoas, erector spinae, and quadratus lumborum muscles), the ratio of the total psoas muscle area to the lumbar spine area (psoas muscle-to-spine ratio) and the ratio of the total muscle area to the lumbar vertebral body area (psoas muscle-to-vertebra ratio). The CT density of these areas was also measured for the evaluation of myosteatosis.

### 2.6. Incorporation of Muscle Depletion and HCC Staging Systems

Because the current HCC staging systems do not include muscle depletion for measuring the nutritional status and the liver reserve of patients with HCC, we combined them to optimize the performance of those systems. In particular, we combined muscle depletion (i.e., muscle-to-spine ratio < 4.42; yes = 1, no = 0) with the CTP score, albumin–bilirubin (ALBI) grade, and BCLC staging to develop new staging systems (Table 2). Patients with CTP scores A, B, and C were assigned 1, 2, and 3 points, respectively. We combined muscle depletion (MD) with the CTP score, ALBI grade, and BCLC stage to obtain the MD–CTP score (range: 1–4, Table 2), MD–ALBI score, and MD–BCLC score, respectively.

### 2.7. Statistical Analysis

Statistical analysis was performed using SPSS version 24 (IBM, Armonk, NY, USA) and Stata software (v15.0, Stata Corp., College Station, TX, USA). The *X*^2^ test or Fisher’s exact test were used to analyze categorical variables, and the Mann–Whitney ranked sum test was used to analyze continuous variables. The diagnostic accuracy of the prognosis score was tested using time-dependent ROC. Sensitivity and specificity were determined using the cutoff point with the highest Youden index (sensitivity + specificity − 1). The OS was analyzed using Kaplan–Meier analysis, followed by the log-rank test. Independent prognostic factors that were possibly related to OS were analyzed. Factors significant in the univariate analysis were included in the adjusted multivariate Cox proportional hazards model for calculating the adjusted hazard ratio (aHR) and the 95% CI.

The discriminatory ability of different models for OS was examined using the Cox proportional hazards model; model fitness (i.e., how the model affects the dependent variable) was assessed using the corrected Akaike information criterion (AICc) [21,22], with a lower AICc value indicating a more explanatory and informative model [23]. We also used the concordance index (C-index), a commonly used metric in survival analysis, to evaluate the performance of predictive models. The C-index can be seen as the fraction of all pairs of individuals whose predicted survival times are correctly ordered [24] and is based on Harrell’s C statistics [25]. A C-index score around 0.70 indicates a good model, whereas a score around 0.50 means random background. For all tests, *p* < 0.05 was considered statistically significant.

### 2.8. Power Analysis

Due to the lack of a power analysis sample size calculation, we applied a post hoc power analysis to evaluate the statistical strength of the results. A post hoc power analysis was conducted to assess our statistical power by using commercially available ClinCalc (ClinCalc LLC., Chicago, IL, USA), online statistic calculators (http://clincalc.com/Stats/Power.aspx (accessed on 1 December 2023)), with a significance level of 5%, and a power of 0.8 [26].

## 3. Results

### 3.1. Baseline Characteristics

The prospective data set of 1072 patients (median age: 63.2 years; 70.8% men) with different stages of HCC who underwent various HCC treatments were consecutively enrolled during the study period (median follow-up: 755 days or 4072.4 person-years). Patients’ baseline clinicodemographic characteristics are summarized in Table 3. Of 1072 patients, 354 (33%) had HBV infection, and 765 (71.4%) had liver cirrhosis; moreover, 405 (37.8%) patients had a tumor size of ≥5 cm. More patients had BCLC stage A (357/1072, 33.4%), Child–Pugh class A (729/1072, 68.0%), and ALBI grade 1 (687/1072, 64.1%).

### 3.2. Clinical Predictors of OS in HCC

**Figure 1:**
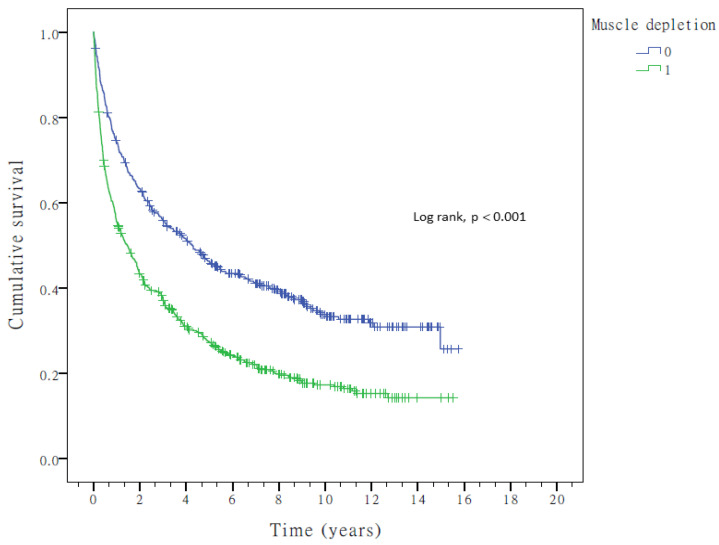
Kaplan–Meier overall survival curves of patients with HCC stratified by muscle depletion.

The median OS was 26.2 months, and 771 (71.9%) patients died during follow-up. The estimated 1-, 3-, and 5-year survival rates were 42.8%, 29.9%, and 23.4%, respectively. Patients with older age (>65 years), HBV or HCV infection, liver cirrhosis, higher AFP level (>20 ng/mL), or larger tumor size (>5 cm) had shorter survival (*p* < 0.01; Table 4). Patients with muscle depletion had shorter survival than those without [1.46 years, 95% CI: 1.12–1.77 vs. 4.29 years, 95% CI: 3.27–5.30, *p* < 0.001] (Figure 1). In multivariate analysis, all these factors, except older age, were independent survival predictors for patients with HCC: HBV infection (aHR: 0.674, 95% CI: 0.571–0.795, *p* < 0.001), HCV infection (aHR: 0.809, 95% CI: 0.676–0.970, *p* = 0.022), liver cirrhosis (aHR: 1.543, 95% CI: 1.303–1.877, *p* < 0.001), larger tumor size > 5 cm (aHR: 1.533, 95% CI: 1.288–1.225, *p* < 0.001), higher AFP level (aHR: 1.704, 95% CI: 1.465–1.982, *p* < 0.001), and muscle depletion (aHR: 1.566, 95% CI: 1.351–1.814, *p* < 0.001).

### 3.3. Noninvasive Liver Functional Reserve Models and OS

The prediction of OS by several liver functional reserve models was examined in patients with HCC. Among them, the BCLC stage, Child–Pugh class, and ALBI grade, which are commonly used in current clinical practice, had higher homogeneity and lower AICc values than other models, indicating a better performance for survival prediction in patients with HCC (Table 5).

### 3.4. Incorporation of Muscle Depletion into Current HCC Staging Systems

**Figure 2:**
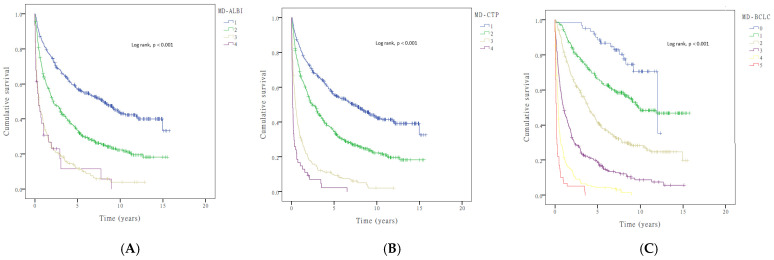
Kaplan–Meier overall survival curves of patients with HCC stratified by the (**A**) MD–ALBI; (**B**) MD–CTP; (**C**) MD–BCLC model scores. ALBI, albumin–bilirubin; BCLC, Barcelona Clinic Liver Cancer; CTP, Child–Turcotte–Pugh; MD, muscle depletion.

**Figure 3:**
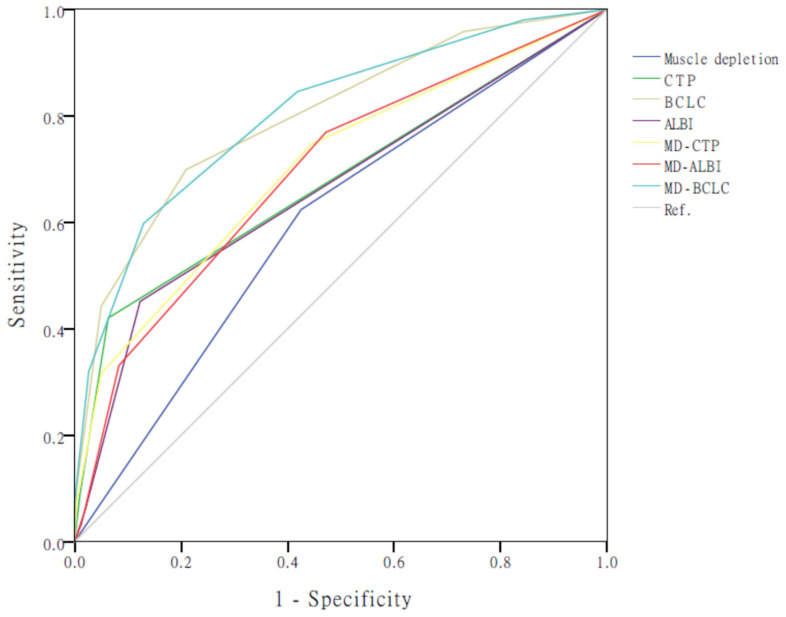
Receiver operating characteristics curve analysis of the various prognostic models. ALBI, albumin–bilirubin; BCLC, Barcelona Clinic Liver Cancer; CTP, Child–Turcotte–Pugh; MD, muscle depletion.

The AUROC of the MD–ALBI, MD–BCLC, and MD–CTP models was better than those of the individual components, with values of 0.694 (95% CI: 0.660–0.728), 0.804 (95% CI: 0.777–0.832), and 0.701 (95% CI: 0.668–0.734), respectively (Figure 2). The MD–BCLC model had better AUROC than that of the MD–CTP model, MD–ALBI model, CTP, and ALBI (all *p* < 0.001) and was an independent survival predictor of patients with HCC (Table 4). In addition, the C-index score of MD-BCLC (0.780) was higher than MD-CTP (0.760) and MD-ALBI (0.753), and both were higher than 0.7, indicating a good model. Furthermore, the MD–BCLC model displayed better performance than the BCLC stage alone for differentiating survival distributions for patients with HCC (*p* < 0.001; post hoc power, 100.0%; Figure 3, Table 4). In particular, it could significantly distinguish the survival risk between patients with BCLC stage B and C compared with BCLC staging (AUROC: 0.86, 95% CI: 0.81–0.91 vs. 0.80, 95% CI: 0.77–0.83).

## 4. Discussion

Muscle depletion is strongly associated with various complications and increased mortality in patients with HCC [27,28]. However, its precise role as a prognostic factor for overall survival (OS) in HCC remains unclear, particularly in terms of its independent predictive value and the mechanisms by which it influences survival outcomes. In this retrospective cohort study, muscle depletion could not only predict the survival of patients receiving various HCC treatments but also improve the discriminatory ability of BCLC staging for HCC.

We observed that their combination—the MD–BCLC model—outperformed other individual and combined models for survival prediction and could identify patients with intermediate or advanced HCC with a high mortality risk. Taken together, our results indicate that the addition of muscle depletion to commonly used noninvasive HCC prognostic models, particularly BCLC staging, improved the discriminatory ability of those models and could identify individuals who may be eligible for more aggressive treatments and close surveillance.

Unlike most noninvasive liver reserve prediction systems, which rely on biochemical parameters, muscle depletion is primarily evaluated radiologically in patients with malignancy. As such, it offers a more objective measurement and has gradually become a widely accepted metric for assessing nutritional and performance status in patients with cancer [29]. Although BCLC staging is the most commonly used system for prognostic prediction and treatment allocation in HCC patients [6], it evaluates liver function using the Child–Pugh classification, which includes some subjective variables. This limits its predictive power, especially in patients with end-stage liver disease requiring transplantation. Consequently, several other noninvasive liver reserve prediction systems have been proposed, with much research focusing on muscle depletion, which provides a continuous metric and offers more refined clinical information compared to the Child–Pugh classification.

In this study, we examined the prognostic value of several noninvasive liver reserve prediction models in 1072 patients with HCC at different stages. We found that BCLC staging had the highest AUROC and homogeneity for survival prediction, suggesting that it is an accurate and reliable tool for predicting OS in HCC patients. Furthermore, incorporating muscle depletion into noninvasive liver reserve prediction models significantly improved their prediction power. Among them, the MD–BCLC combination model demonstrated the highest AUROC, indicating the best accuracy for predicting survival in these patients. Stratified analysis further revealed that the MD–BCLC model enhanced the survival risk prediction for patients with intermediate or advanced stages of HCC. These patients are more suitable for early conversion to new, combined, or adjuvant/neoadjuvant treatments, especially when they are refractory to standard care options. Further clinical trials are needed to validate this finding.

In Taiwan, HCC patients are treated according to internationally recommended guidelines (e.g., AASLSD, EASL, APASL). As part of the treatment process, all patients with HCC undergo screening by a multidisciplinary team, which includes radiologists, surgeons, hepatologists, and oncologists. This team assesses the clinical diagnosis, tumor resectability, and staging, and discusses treatment options with patients and their families. Based on these discussions, patients select their preferred treatment. Most patients with HCC receive treatment according to their BCLC stage: surgery or RFA or transplantation for those with stage A, TACE for those with stage B, systemic therapy for those with stage C, and the best supportive care for those with stage D. In this study, we compared the discriminatory ability of the MD–BCLC model with BCLC staging alone.

Approximately 50% of the patients in our study were HBV or HCV positive. In Taiwan, the National Health Insurance (NHI) reimburses treatment for HBV- and HCV-related HCC. As a result, nearly all HCC patients with HBV or HCV infection in this study received antiviral therapy. Therefore, patients with HBV or HCV infection likely had better survival outcomes than those without infection, which may have influenced the results.

This study has some limitations. First, its retrospective design could lead to selection bias and the extraction of incomplete data; in addition, other factors may also have confounded survival outcomes. Consequently, our results should be cautiously generalized and attributed to causality. Second, this study did not examine the effect of antiviral therapy, which inevitably affects patient prognosis. However, because Taiwan’s National Health Insurance covers antiviral treatments for patients with HCC having HBV or HCV infection, almost all patients with HBV- or HCV-related HCC received antiviral therapy in this study. Therefore, patients with HBV or HCV infection likely had better survival than those without infection in our analysis. Finally, although this study involved patients with different stages of HCC who received various standards of care, the sample size of this single-center study is inadequate to clarify the effect of the different states of the disease or treatment scenarios. Future larger-scale multicenter prospective studies with fine-tuned subgroup analyses are warranted.

## 5. Conclusions

This study highlights the significant role of MD as an independent prognostic factor in HCC. By incorporating MD into the BCLC staging system, this study demonstrates an improved OS prediction for HCC patients. The findings suggest that the MD-BCLC model improves the precision of survival predictions, particularly for patients with intermediate or advanced stages of HCC, who may benefit from more aggressive treatment approaches. This model enables better risk stratification and more personalized treatment decisions, helping identify high-risk patients who may benefit from more aggressive treatments.

Future research should focus on multi-center validation, the prospective evaluation of the MD-BCLC model, treatment-specific subgroup analyses, cost-effectiveness analysis of MD measurements, and the exploration of intervention strategies tailored to MD status to improve patient outcomes. Further studies are needed to validate its role in routine clinical practice.

## Figures and Tables

**Table 1:** Formula and grading of 14 noninvasive liver functional reserve models.

Noninvasive Blood Testing for Liver Reserve Makers	Formula
ALBI, Grade 1/2/3 (<−2.6/−2.6–≤−1.39/>−1.39)	(log(Bilirubin[μmol/L]) × 0.66) + (Albumin[g/L] × −0.085)
APRI, Grade 1/2/3 (0.5/0.5–1.5/>1.5)	[(AST/upper limit of normal)/Platelet Count (10^9^/L)] × 100
CDS, Grade 1/2/3 (<4/4–7/>7)	Platelet count (×10^9^/L): >340 = 0; 280–339 = 1; 220–279 = 2; 160–219 = 3; 100–159 = 4; 40–99 = 5; <40 = 6 ALT/AST ratio: >1.7 = 0; 1.2–1.7 = 1; 0.6–1.19 = 2; <0.6 = 3 INR: <1.1 = 0; 1.1–1.4 = 1; >1.4 = 2 CDS is the sum of the above (possible value 0–11).
BCLC, Stage 0, A, B, C, D	As reference [21].
CTP scores A, B, C	Encephalopathy: none = 1, grade 1 or 2 = 2, grade 3 or 4 = 3; ascites: none = 1, mild to moderate = 2, severe = 3; bilirubin(mg/dL): <2 = 1, 2–3 = 2, >3 = 3; albumin(g/dL): >3.5 = 1, 2.8–3.5 = 2, <2.8 = 3 PT; sec (INR): <4 (1.7) = 1, 4–6 (1.7–2.3) = 2, >6 (>2.3) = 3
FIB-4 index, Grade 1/2/3 (<1.45/1.45–3.25/>3.25)	(Age[years] × AST[U/L])/(platelet [10^9^] × ALT[U/L]^1/2^)
GUCI, Grade 1/2/3 (<0.5/0.5–1.56/>1.56)	[AST/TOPNORMAL AST] × INR × 100/(Platelets × 10^9^)
Lok index, Grade 1/2/3 (<0.5/0.5–0.8/>0.8	Lok Index = e^(LogOddsLok)^/(1 + e^(LogOddsLok)^) Log Odds Lok = (1.26 × AST/ALT) + (5.27 × INR)− (0.0089 × Platelets × 10^9^) − 5.56
MELD, Grade 1/2/3 (<8/8–12/>12)	10 × ((0.957 × ln(Creatinine)) + (0.378 × ln(Bilirubin)) + (1.12 × ln(INR))) + 6.43
PALBI, Grade1/2/3 (≤−2.53, −2.53 and ≤−2.09, >−2.09)	(2.02 × log_10_ bilirubin) − [0.37 × (log_10_ bilirubin(umol/L))^2^] − 0.04 × albumin (g/L) − 3.48 × log_10_ platelets(10^9^/L) + 1.01 × (log_10_ platelets(10^9^/L))^2^
King’s score (<7.6/7.6–16.7/16.7)	Age × AST × INR/[platelets (10^9^/L)]
Psoas muscle-to-spine ratio	4.42 (ROC curve cut point)
Psoas muscle-to-vertebra ratio	2.32 (ROC curve cut point)
CT density of Psoas muscle area	37.2 (ROC curve cut point)

ALBI, albumin–bilirubin; APRI, aspartate aminotransferase-to-platelet ratio; BCLC, Barcelona Clinic Liver Cancer; CDS, cirrhosis discriminant index; CTP, Child–Turcotte–Pugh; CT, computed tomography; FIB-4, fibrosis index based on 4 factors; GUCI, Goteborg University Cirrhosis Index; MELD, Model for end-stage liver disease; PALBI, platelet–albumin–bilirubin.

**Table 2:** CTP score, ALBI grade, BCLC stage, and muscle depletion grading systems alone or in combination.

Parameters	Scores					
CTP score	1	2	3	_	_	_
ALBI grade	1	2	3	_	_	_
BCLC stage	0	1	2	3	4	_
Muscle depletion	0	1	_	_	_	_
MD–CTP score	1	2	3	4	_	_
MD–ALBI grade	1	2	3	4	_	_
MD–BCLC stage	0	1	2	3	4	5

ALBI, albumin–bilirubin; BCLC, Barcelona Clinic Liver Cancer; CTP, Child–Turcotte–Pugh; MD, muscle depletion. Muscle depletion was defined as the psoas muscle-to-spine ratio < 4.42.

**Table 3:** Patients’ baseline clinicodemographic characteristics.

Variables	Patients (*n* = 1072)
Age (Mean ± SD)	63.2 ± 12.8
Sex, n (%)	
Male	759 (70.8)
Female	313 (29.2)
HBV infection, n (%)	354 (33.0)
HCV infection, n (%)	268 (25.0)
HBV/HCV co-infection, n (%)	28 (2.6)
Smoking, n (%)	196 (18.3)
Alcohol drinking, n (%)	65 (6.1)
Liver cirrhosis, n (%)	765 (71.4)
Tumor size (≤5 cm/>5 cm), n (%)	667 (62.2)/405 (37.8)
Psoas muscle-to-spine ratio (Mean ± SD)	4.4 ± 1.5
Psoas muscle-to-vertebra ratio (Mean ± SD)	2.3 ± 0.8
Laboratory values (Mean ± SD)	
AST (IU/L)	93.0 ± 196.3
ALT (IU/L)	67.2 ± 122.5
Albumin (g/dL)	3.3 ± 0.8
Total bilirubin (mg/dL)	1.5 ± 2.6
Creatinine (mg/dL)	1.4 ± 5.6
Platelets (1000/μL)	162.0 ± 99.0
INR	1.1 ± 0.2
BCLC stage (0/A/B/C/D), n (%)	113 (10.5)/357 (33.4)/246 (22.9)/280 (26.1)/76 (7.1)
CTP score (A/B/C), n (%)	729 (68.0)/272 (25.4)/71 (6.6)
ALBI grade (1/2/3), n (%)	687(64.1)/333 (31.1)/52 (4.9)
APRI grade (1/2/3), n (%)	223 (20.8)/465 (43.4)/384 (35.8)
CDS grade (1/2/3), n (%)	87 (8.1)/709 (66.1)/276 (25.7)
Child–Pugh class (1/2/3), n (%)	729 (68.0)/272 (25.4)/71 (6.6)
FIB-4 grade (1/2/3), n (%)	134 (12.5)/322 (30.0)/616 (57.5)
GUCI grade (1/2/3), n (%)	193 (18.0)/457 (42.6)/422 (39.4)
King’s score (1/2/3), n (%)	91 (8.5)/218 (20.3)/763 (71.2)
Lok index grade (1/2/3), n (%)	381 (35.5)/351 (32.7)/340 (31.7)
MELD score (1/2/3), n (%)	4 (0.4)/54 (5.0)/1014 (94.6)
PALBI grade (1/2/3), n (%)	25 (2.3)/50 (4.7)/997 (93.0)
Psoas muscle-to-spine ratio (>4.42/≤4.42), n (%)	463 (43.2)/609 (56.8)
Psoas muscle-to-vertebra ratio (>2.32/≤2.32), n (%)	473 (44.1)/599 (55.9)
CT density of psoas muscle area (>37.2/≤37.2), n (%)	487 (45.4)/585 (54.6)

ALBI, albumin–bilirubin; ALT, alanine aminotransferase; APRI, aspartate aminotransferase-to-platelet ratio; AST, aspartate aminotransferase; BCLC, Barcelona Clinic Liver Cancer; CDS, cirrhosis discriminant index; CTP, Child–Turcotte–Pugh; CT, computed tomography; FIB-4, fibrosis index based on 4 factors; GUCI, Goteborg University Cirrhosis Index; HBV, hepatitis B virus; HCV, hepatitis C virus; INR, international normalized ratio; MELD, model for end-stage liver disease; PALBI, platelet–albumin–bilirubin; SD, standard deviation.

**Table 4:** Univariate and multivariate analyses of factors associated with overall survival.

Overall Survival	N	Univariate Analysis			Multivariate Analysis		
		HR	95%CI	*p*-Value	HR	95%CI	*p*-Value
Age (>65/≤65)	467/605	1.207	1.047–1.392	0.009			
Sex (male/female)	759/313	0.996	0.853–1.164	0.963			
HBsAg (positive/negative)	354/718	0.591	0.505–0.691	<0.001	0.674	0.571–0.795	<0.001
Anti-HCV (positive/negative)	268/804	0.750	0.632–0.889	0.001	0.809	0.676–0.970	0.022
Alcohol (yes/no)	65/1007	1.120	0.831–1.510	0.456			
Smoke (yes/no)	196/876	0.925	0.765–1.118	0.419			
DM (yes/no)	195/877	0.916	0.763–1.100	0.349			
Liver cirrhosis (yes/no)	765/307	1.444	1.226–1.702	<0.001	1.543	1.303–1.827	<0.001
Tumor size (>5 cm/≤5 cm)	405/667	3.502	3.022–4.058	<0.001	1.533	1.288–1.825	<0.001
Alpha-fetoprotein (>20/≤20 ng/mL)	594/478	2.043	1.763–2.367	<0.001	1.704	1.465–1.982	<0.001
Muscle depletion (MD: psoas muscle-to-spine ratio > 4.42 vs. ≤4.42)	609/463	1.737	1.500–2.011	<0.001	1.566	1.351–1.814	<0.001
MD–BCLC stage							
0	62	1			1		
1	232	2.178	1.267–3.743	<0.001	2.007	1.167–3.454	<0.001
2	278	4.305	2.543–7.286	<0.001	3.461	2.038–5.877	<0.001
3	245	9.807	5.797–16.592	<0.001	6.238	3.638–10.696	<0.001
4	196	20.938	12.303–35.632	<0.001	12.881	7.450–22.269	<0.001
5	59	43.949	24.696–78.213	<0.001	29.553	16.349–53.421	<0.001

**Table 5:** Predictive accuracy of overall survival in 14 noninvasive liver functional reserve models.

Overall Survival	Corrected Akaike Information Criteria (AIC)	Homogeneity (Wald x2)	Concordance Index (C-Index)
ALBI grade	5557.464	97.244	0.749
APRI grade	5621.803	32.905	0.733
BCLC stage	5466.796	192.023	0.782
CDS grade	5618.206	36.503	0.726
CTP score	5542.581	112.127	0.762
FIB-4 grade	5627.292	27.416	0.729
GUCI grade	5608.640	46.069	0.737
King’s score	5628.484	26.224	0.730
Lok index grade	5568.016	86.692	0.743
MELD score	5652.365	2.344	0.720
PALBI grade	5653.887	0.822	0.720
Psoas muscle-to-spine ratio	5628.330	24.329	0.726
Psoas muscle-to-vertebra ratio	5645.909	6.750	0.724
CT density of psoas muscle area	5648.150	4.509	0.721

ALBI, albumin–bilirubin; APRI, aspartate aminotransferase-to-platelet ratio; BCLC, Barcelona Clinic Liver Cancer; CDS, cirrhosis discriminant index; CTP, Child–Turcotte–Pugh; CT, computed tomography; FIB-4, fibrosis index based on 4 factors; GUCI, Goteborg University Cirrhosis Index; MELD, model for end-stage liver disease; PALBI, platelet–albumin–bilirubin.

## Data Availability

Data can be found within the article.
